# Evaluation of tocilizumab therapy in patients with rheumatoid arthritis based on FDG-PET/CT

**DOI:** 10.1186/1471-2474-15-393

**Published:** 2014-11-22

**Authors:** Koichi Okamura, Yukio Yonemoto, Chisa Okura, Tetsuya Higuchi, Yoshito Tsushima, Kenji Takagishi

**Affiliations:** Department of Orthopaedic Surgery, Gunma University Graduate School of Medicine, 3-39-22, Showamachi, Maebashi, Gunma 371-8511 Japan; Department of Diagnostic Radiology and Nuclear Medicine, Gunma University Graduate School of Medicine, Maebashi, Gunma Japan

**Keywords:** Rheumatoid arthritis, Interleukin-6, Positron emission tomography, SUV, Synovitis, Tocilizumab

## Abstract

**Background:**

Positron emission tomography (PET) with 2-[18F]-fluoro-2-deoxy-D-glucose (^18^F-FDG) can detect the presence of synovitis in rheumatoid arthritis (RA) patients. The aim of this study was to investigate whether the findings of FDG-PET matched the conventional assessments of the disease activity score (DAS) 28, DAS28-CRP, simplified disease activity index (SDAI) and clinical disease activity index (CDAI) in RA patients receiving tocilizumab (TCZ) therapy.

**Methods:**

Seventeen RA patients treated with TCZ were assessed. FDG-PET was performed at baseline and three and six months after the initiation of TCZ therapy. The maximum SUV (SUVmax) of the bilateral shoulder, elbow, wrist, hip, knee and ankle joints were added together (total SUV) and were used to assess the degree of FDG uptake as a representative parameter. The correlations between the ΔSUV and the difference in the clinical parameters at baseline and at each observation period, and the differences in each clinical parameters, were assessed.

**Results:**

The ΔSUV, the differences in the total SUV at baseline and at three/six months after starting treatment positively correlated with the ΔDAS28 (r = 0.615 p = 0.009/ r = 0.775 p < 0.001), ΔDAS28-CRP (r = 0.696, p = 0.002/ r = 0.828, p < 0.001), ΔSDAI (r = 0.652, p = 0.005/ r = 0.686, p = 0.002) and ΔCDAI (r = 0.662, p = 0.004/ r = 0.711, p = 0.001) for each period. The total SUV was significantly decreased at three and six months after the initiation of TCZ (p < 0.05).

**Conclusions:**

A reduction in the FDG uptake was observed at three and six months after the initiation of TCZ therapy. The disease activity estimated on FDG-PET/CT matched the conventional parameters following the TCZ therapy in RA patients.

**Electronic supplementary material:**

The online version of this article (doi:10.1186/1471-2474-15-393) contains supplementary material, which is available to authorized users.

## Background

In recent years, the treatment of rheumatoid arthritis (RA) has rapidly advanced, and many biologic drugs, including tumor necrosis factor (TNF) inhibitors, are now available [1]. Newly developed biologics have changed the therapeutic strategy for treating RA. A humanized anti-interleukin-6 receptor (anti-IL-6R) antibody, tocilizumab (TCZ), is one of these new drugs [2, 3].

Previous studies have reported that the C-reactive protein (CRP) level and erythrocyte sedimentation rate (ESR) immediately decrease after the initiation of TCZ therapy [4], and almost all physicians are thought to agree that this phenomenon occurs. Therefore, some authors have proposed that the Disease Activity Score in 28 Joints (DAS28) is an inappropriate marker for assessing the effectiveness of TCZ therapy for patients with RA [5–7].

In addition, other scoring systems, such as the DAS28-CRP and simplified disease activity index (SDAI), which has been recommended for assessing the disease activity of RA patients [8], include the effects of CRP. Although the European League Against Rheumatism recommends early intervention to achieve a maximum effect [8], clinical physicians are sometimes encounter an patient whose serum CRP level have decreased soon after the initiation of TCZ, regardless of the unchanged condition of the RA.

Positron emission tomography (PET) with 2-[^18^F]-fluoro-2-deoxy-D-glucose (^18^F-FDG) can be used to evaluate the metabolic activity of RA [9–12]. RA patients display pathological changes, including synovitis, pannus formation and bone erosion. Because ^18^F-FDG PET can be used to precisely recognize an increase in synovitis in affected joints, imaging studies with ^18^F-FDG PET have been performed to assess the metabolic activity of synovitis in RA patients and evaluate the disease activity of RA [13–17]. In addition, PET enables the quantitative measurement of RA joints using the standardized uptake value (SUV). The FDG uptake represented by the SUV value in the inflamed RA joints reflects the disease activity [18].

Hence, in the present study, we evaluated whether the findings of FDG-PET matched the conventional assessments of the DAS28, DAS28-CRP, SDAI and clinical disease activity index (CDAI) at three and six months after initiating TCZ therapy in RA patients.

## Methods

### Patients and methods

The Institutional Review Board of Gunma University Hospital approved the protocol for this study, and informed consent was obtained from each patient. Seventeen patients (5 males, 12 females; average age: 59.9±11.7 (30–82) years) were enrolled in this study.

All patients were diagnosed according to the American College of Rheumatology (ACR) criteria revised in 1987 [19], and all had a history of a clinically inadequate response to previous treatment with non-biological disease modifying antirheumatic drugs (DMARDs), including methotrexate (MTX) and to biological agents, including TNF inhibitor monotherapy. Therefore, all patients were candidates for treatment with TCZ and TCZ therapy was performed as part of their standard care. During the six months prior to the baseline and during the six months after stating TCZ therapy, there were no changes in the therapies used for RA in the patients under TCZ therapy except for the use of TCZ.

The average disease duration was 12.7±10.4 (1–41) years. The number of RA patients in each stage and class according to the Steinbrocker classification was as follows: stage I/II/III/IV: 2/0/5/10; and class 1/2/3/4: 2/9/5/1. MTX was administered in 13 patients (76.5%), at an average dose of 9.3 (4–16) mg/week, tacrolimus (TAC) was administered in five patients (29.4%), at an average dose of 2.30 (1.5 - 3.0) mg/day, and prednisolone (PSL) was prescribed in 11 patients (64.7%), at an average dose of 5.2 (1–15) mg/day (Table 1).

**Table 1:** **Characteristics of the enrolled patients**

	Total n = 17
Age (years)	59.9 ± 11.7 (30–82)
Sex (male/female)	5/ 12
Disease duration (years)	12.7 ± 10.4 (1–41)
Steinbrocker Stage (I/II/III/IV)	2/0/5/10
Steinbrocker Class (1/2/3/4)	2/9/5/1
MTX (%)/dose of MTX (mg/week)	76.5/ 9.3 ± 3.9 (4.0 - 16.0)
PSL (%)/dose of PSL (mg/day)	64.7/ 5.2 ± 3.7 (1.0 - 15.0)
Tacrolimus (%)/dose of tacrolimus (mg/day)	29.4/ 2.3 ± 0.7 (1.5 - 3.0)

MTX: methotrexate, PSL: prednisolone.

After undergoing a baseline assessment with whole-body ^18^F-FDG PET combined with computed tomography (CT) (FDG-PET/CT), the subjects received TCZ therapy. FDG-PET/CT was performed at three and six months after the initiation of treatment. Clinical parameters, including the ESR and serum CRP and matrix metalloproteinase-3 (MMP-3) concentrations, were obtained each month from baseline to six months after the initiation of TCZ. The degree of disease activity was assessed using the DAS28, DAS28-CRP, SDAI and CDAI. The DAS28 score was calculated based on the results of the 28 tender joint count (TJC), 28 swollen joint count (SJC) and ESR, as well as the patient’s global health, as represented by the visual analog scale (P-VAS). The DAS28-CRP score was calculated using the TJC, SJC, CRP and P-VAS [20]. The SDAI score was calculated as the total of the TJC, SJC, CRP, P-VAS scores and the evaluator global disease activity determined according to the visual analog scale (E-VAS) [21]. The CDAI score was calculated as the total of the TJC, SJC, P-VAS and E-VAS scores [22].

### PET images

Whole-body PET scans were performed 60 minutes following the intravenous injection of ^18^F-FDG (5 MBq/kg) after the patient had fasted for more than six hours. Data acquisition was carried out in 3D mode using a PET-CT scanner (Biograph 16, Siemens Medical Solutions, Inc.). The patients were scanned from the head to the toe in the arms-down position, according to a previous report [17]. The PET images were interpreted by the experienced nuclear physicians, who recorded an increased FDG uptake in the bilateral shoulder, elbow, wrist, hip, knee and ankle joints.

### Data analysis

For the semiquantitative analysis of the PET images, functional images of the SUV were produced using attenuation-corrected transaxial images, the injected dose of FDG, patient’s body weight and cross-calibration factor between PET and the dose calibrator. The SUV was defined as follows:

SUV = Radioactive concentration in the region of interest (ROI) [MBq/g]/Injected dose [MBq]/Patient’s body weight [g].

ROIs were manually drawn, and the ROI analyses were conducted by a nuclear physician with the assistance of CT images of the areas. The maximum SUV in the ROI was used as a representative value for the assessment of the FDG uptake. The therapeutic response was evaluated based on the changes in the total value of the maximum SUV of the affected joints (total SUV) and the DAS28, DAS28-CRP, SDAI, CDAI, ESR, CRP and MMP-3 scores. We used either all 12 joints evaluated on FDG PET or eight (bilateral shoulder, elbow, wrist and knee) joints represented in the DAS28, SDAI and CDAI to calculate the total SUVmax. The delta SUV (ΔSUV), delta DAS28 (ΔDAS28), delta DAS28-CRP (ΔDAS28-CRP), delta SDAI (ΔSDAI) and delta CDAI (ΔCDAI) were defined as the difference between the baseline value and that obtained three or six months after the initiation of therapy. For example, ΔSUV_3M-0M_ means the difference in the SUVmax between baseline and three months. In the following Results section, Δ indicates the difference in the values between the observation periods.

### Statistical analysis

Spearman’s rank correlation test was applied to evaluate the correlations between the different parameters recorded in this study. Based on a power analysis (G*power 3.1, http://www.gpower.hhu.de/), the estimated total sample size was at least 16 patients (correlation ρ H1 = 0.65, α = 0.05, β = 0.20). The treatment response was evaluated on both a patient and joint basis. Wilcoxon’s signed-rank sum test was used to assess the differences in the effects of treatment. All of the results are expressed as the means±SD. The IBM SPSS Statistics 21 software program (International Business Machines Corp., New York, US) was used for the analysis, and values of P < 0.05 were considered to be statistically significant. To assess multiple correlations, we used the Bonferroni correction [23], and the correlation coefficient was calculated.

## Results

**Figure 1:**
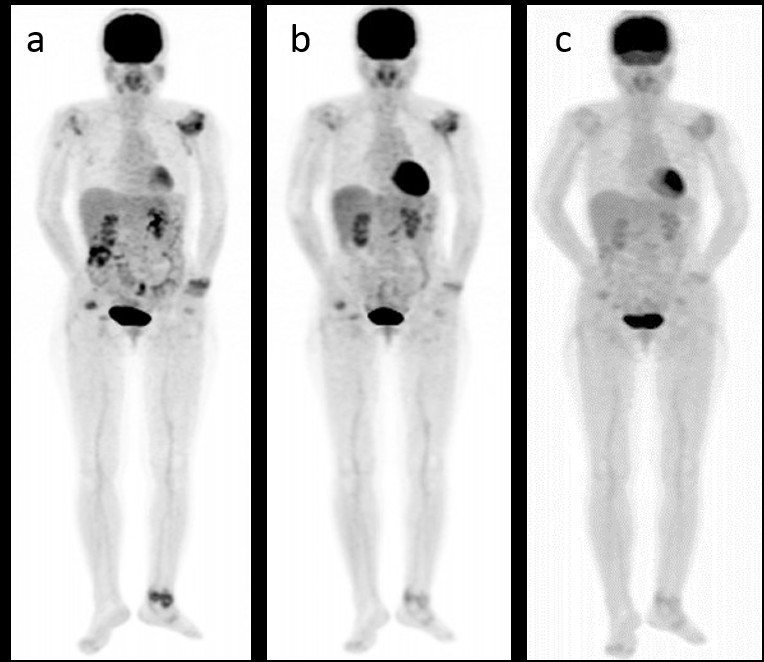
**Images of FDG-PET obtained under TCZ therapy. (a)** Whole-body ^18^F-FDG-PET/CT was performed before **(a)** at three **(b)** and six months **(c)** after the initiation of TCZ therapy. The FDG uptake in the affected joints had decreased. TCZ: tocilizumab. The contrasts of the PET images were adjusted on the basis of the liver density.

Figure 1 shows the PET-CT images in a typical case, both before therapy and at the follow-up examinations.

**Figure 2:**
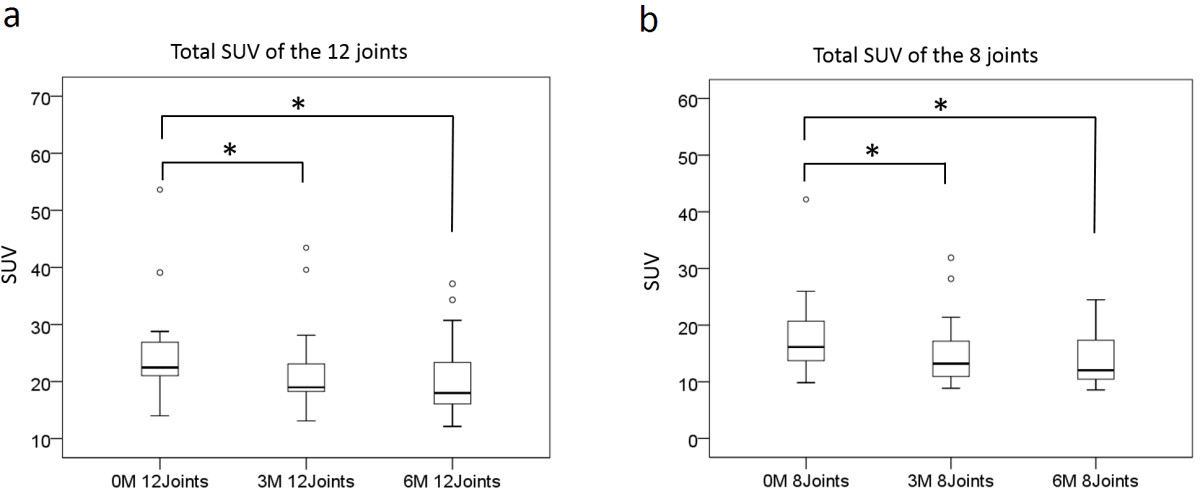
**Total SUV values of the affected joints.** The total SUV values of 12 joints **(a)** and eight joints **(b)** are shown. The total SUV values were significantly decreased at three months and six months compared to baseline. The bars within the squares indicate the medians of the total SUV values. The median and range of each observation were the following: **(a)** 12 joints; 22.5 (13.9 – 53.6), 19.0 (13.1 – 43.5) and 18.0 (12.2 – 37.1). **(b)** 8 joints; 16.1 (9.9 – 42.2), 13.2 (8.9 – 31.9) and 12.0 (8.6 – 24.5). *indicates a significant difference compared with the value observed at baseline (Wilcoxon signed rank-sum test, p < 0.05).

The responses of the RA patients to TCZ therapy evaluated based on the clinical parameters are shown in Table 2. The disease activity, as assessed according to the composite measurements, DAS28-CRP and SDAI, which included the CRP level, was significantly decreased at three months and six months. Similarly, the CDAI, which did not include the serum CRP level, was also decreased at three months and six months after the initiation of TCZ treatment. The serum MMP-3 level was also significantly decreased at three and six months after starting treatment.In addition, the total SUV values decreased at three months (18.1±7.9/15.3±6.6) and at six months (21.8±8.3/14.5±8.6) compared to those observed at baseline (24.8±9.5/20.8±7.7) (12/8 joints, respectively) (Figures 2a, 2b).

**Table 2:** **Parameters for all patients before and after TCZ treatment**

	Before treatment	After 3M treatment	After 6M treatment
	All patients	All patients	All patients
	Mean ± SD (range)	Mean ± SD (range)	Mean ± SD (range)
DAS28	4.7±1.4 (2.5-8.9)	3.2±1.5 (1.3-6.3)*	3.1±1.6 (0.9-6.7)*
DAS28-CRP	3.2±1.4 (1.3-8.9)	2.4±1.3 (1.0-5.2)*	2.4±1.4 (1.0-5.2)*
SDAI	20.2±16.6 (3.2-75.1)	10.4±10.8 (0.7-40.9)*	11.7±13.3 (0.1-42.8)*
CDAI	18.1±15.4 (2.8-68)	9.8±10.0 (0.7-39)*	11.1±13.0 (0.1-42.8)*
ESR (mm/h)	67.8±33.9 (14–122)	23.4±26.0 (3–107)*	20.9±25.0 (2–98)*
CRP (mg/dl)	2.16±2.72 (0.11-7.10)	0.60±1.48 (0.01-5.73)*	0.53±1.55 (0.00-6.25)*
MMP-3 (ng/ml)	302.6±254.2 (28.4-792.6)	150.1±153.4 (32.1-600.7)*	139.6±152.3 (10.0-545.4)*
Tender joints count	3.6±5.9 (0–24)	2.5±3.6 (0–13)	2.9±5.0 (0–18)
Swollen joints count	5.7±5.8 (0–24)	3.1±3.6 (0–15)*	3.8±4.9 (0–15)
P-VAS	39.9±29.0 (0–100)	22.5±24.1 (0–70)*	23.0±24.1 (0–71)*
E-VAS	47.9±26.9 (0–100)	19.4±15.9 (0–55)*	20.5±21.8 (1–77)*

The data are presented as the mean (S.D.) (range). The average for each parameter was calculated before treatment and at follow-up.

*indicates a significant difference compared with the value observed at baseline (p < 0.05). P-VAS: patient global disease activity represented by the visual analog scale. E-VAS: evaluator global disease activity represented by the visual analog scale.

According to the correlation analyses, the ΔSUV_3M-0M_ values were positively correlated with the ΔDAS28_3M-0M_ (r = 0.615, p = 0.009/ r = 0.610, p = 0.009), ΔDAS28-CRP_3M-0M_ (r = 0.696, p = 0.002/ r = 0.723, p = 0.001), ΔSDAI_3M-0M_ (r = 0.652, p = 0.005/ r = 0.652, p = 0.005/ r = 0.637, p = 0.006) and ΔCDAI_3M-0M_ (r = 0.662, p = 0.004/ r = 0.640, p = 0.006) values (12/8 joints, respectively). There were also significant correlations between the ΔSUV_6M-0M_ values and the ΔDAS28_6M-0M_(r = 0.775, p < 0.001/r = 0.749, p = 0.001), ΔDAS28-CRP_6M-0M_(r = 0.828, p < 0.001/r = 0.775, p < 0.001), ΔSDAI_6M-0M_(r = 0.686, p = 0.002/r = 0.623, p = 0.008) and ΔCDAI_6M-0M_(r = 0.711, p = 0.001/r = 0.686, p = 0.002) values (12/8 joints, respectively) (Table 3).

**Table 3:** **Correlations between the ΔSUV and the clinical parameters**

		3 months			
		ΔDAS28	ΔDAS28-CRP	ΔSDAI	ΔCDAI
ΔSUV (12 joints)	r	0.615*	0.696*	0.652*	0.662*
	p	0.009	0.002	0.005	0.004
ΔSUV (8 joints)	r	0.61*	0.723*	0.637*	0.64*
	p	0.009	0.001	0.006	0.006
		**6 months**			
		Δ**DAS28**	Δ**DAS28-CRP**	Δ**SDAI**	Δ**CDAI**
ΔSUV (12 joints)	r	0.775*	0.828*	0.686*	0.711*
	p	<0.001	<0.001	0.002	0.001
ΔSUV (8 joints)	r	0.749*	0.775*	0.623*	0.686*
	p	0.001	<0.001	0.008	0.002

The ΔSUV (12/8 joints), the difference in the total SUVmax of the affected 12 or eight (bilateral shoulder, elbow, wrist and knee) joints before and after treatment, was significantly correlated with the ΔDAS28, ΔDAS28-CRP, ΔSDAI and ΔCDAI. *indicates a significant correlation following the Bonferroni correction.

The results of a joint-based analysis of the 12 joints in each of the 17 RA patients are shown in Table 4. We evaluated the changes in the presence of tender and swollen joints and the SUV values of the affected joints before and after TCZ therapy. Consequently, joint tenderness was found to have significantly decreased in the right wrist and bilateral knees at three months and in the left wrist at six months after beginning TCZ treatment. Joint swelling was also found to have decreased in the bilateral wrists at six months after treatment, when evaluated on an individual joint basis. In addition, the SUV values were significantly decreased in the bilateral knees at three months and in the left elbow and bilateral knees at six months in each joint.

**Table 4:** **Results of a joint-based analysis**

	TJC	SJC	SUV	TJC	SJC	SUV	TJC	SJC	SUV
	(0M)	(0M)	(0M)	(3M)	(3M)	(3M)	(6M)	(6M)	(6M)
Rt shoulder	1	0	1.71±1.11	2	0	1.68±0.76	1	0	1.65±0.60
Lt shoulder	0	0	2.06±1.31	1	0	2.06±1.20	1	0	2.11±1.07
Rt elbow	1	2	1.63±1.05	3	3	1.48±0.77	5*	4	1.46±0.58
Lt elbow	2	3	2.05±1.50	5	4	1.70±1.15	5	5	1.64±1.06*
Rt wrist	7	9	2.69±1.24	2*	8	2.30±1.44	3	3*	1.92±1.11
Lt wrist	6	8	2.49±1.44	2	4	2.23±1.57	1*	2*	1.96±1.17
Rt knee	7	9	2.90±1.89	2*	4	1.98±0.82*	4	5	1.89±0.90*
Lt knee	7	7	2.58±1.82	1*	4	1.90±1.22*	2	5	1.84±1.05*
Rt hip	0	0	1.47±0.50	0	0	1.53±0.36	0	1	1.55±0.50
Lt hip	1	0	1.54±0.59	0	0	1.56±0.33	0	1	1.43±0.43
Rt ankle	4	5	1.95±1.21	3	3	1.67±0.99	4	3	1.55±0.91
Lt ankle	5	7	1.95±1.21	4	3	1.75±1.08	5	5	1.79±0.96

The data are presented as the mean ± S.D.. *indicates a significant difference compared with the value observed at baseline (Wilcoxon signed rank-sum test, p < 0.05).

## Discussion

TCZ blocks IL-6 receptors and directly inhibits the production of CRP, thus leading to a decrease in the ESR. Therefore, it is possible that combined assessments of the CRP and ESR values may overestimate the effects of TCZ treatment. Based on this background, we suspected that the conventional clinical assessments of the effects of TCZ are inaccurate.

Funakoshi et al. demonstrated that the remission rate measured according to the CDAI, which does not include the serum CRP level, is approximately half that measured using the DAS28 [5]. On the other hand, Kaneko et al. recently reported that a decrease in the CRP level at 12 weeks after the initiation of TCZ therapy predicts a decrease in the disease activity at 52 weeks [6]. Therefore, we estimated that the suppression of synovitis in RA joints under TCZ therapy occurs at around 12 weeks after the initiation of treatment.

FDG-PET utilizes molecular imaging to obtain images, not of the morphology, but of the metabolism of cells. Okamura et al. evaluated the response to treatment with anti-TNF drugs in RA patients using FDG-PET/CT, and suggested that a semiquantitative method employing the SUV on FDG-PET/CT is useful for assessing the efficacy of biological treatment in patients with RA [17]. Therefore, in this study, we evaluated the effects of TCZ therapy three and six months after the initiation of treatment using the SUVmax of FDG-PET/CT semiquantitatively. To the best of our knowledge, this is the first study to evaluate the efficacy of TCZ therapy using FDG-PET/CT.

In the present study, the total SUV values (12/8 joints) were decreased at three months and at six months compared to those observed at baseline, and the ΔSUV positively correlated with the differences in the disease activity determined according to composite measurements, with or without the CRP level, from baseline to three and six months, respectively. These results indicate that inflammatory synovitis was suppressed in the RA joints at both three and six months after starting TCZ treatment, and that the composite measurements conventionally used by rheumatologists, with or without the serum CRP level or ESR, properly reflect the disease activity in RA patients at three months and six months after the initiation of TCZ therapy.

The precise mechanism(s) underlying the blockage of FDG uptake in synovial cells under biological treatment remain unclear. One potential mechanism is a decrease in the number of synovial cells in inflamed joints due to the effects of biological therapy. IL-6 is likely involved in synovial cell proliferation in the synovium in RA patients in cooperation with sIL-6R [24], and the intraperitoneal administration of the humanized anti-human IL-6R antibody significantly reduces the number of infiltrating inflammatory cells in the implanted synovium [25]. Therefore, the inhibition of IL-6 by TCZ may reduce the number of inflammatory cells in the synovium, and thus would decrease the corresponding FDG uptake.

In addition, IL-6 regulates the expression of vascular endothelial growth factor (VEGF) [26] and intercellular adhesion molecule 1 (ICAM-1) [27], which induces pannus formation in RA joints. It has also been reported that proinflammatory cytokines, as well as hypoxia, contribute to the ^18^F-FDG uptake by cells involved in pannus formation in patients with RA [28]. Therefore, the suppression of neoangiogenesis and cellular infiltration may also reduce the FDG uptake in affected joints.

Although the precise pathological mechanisms underlying the features of the RA synovium observed on ^18^F FDG-PET have not been fully elucidated, a significant decrease in the serum MMP-3 level, which is thought to primarily reflect the volume of the RA synovium, was observed at both three and six months after the initiation of TCZ treatment in the present study. These results indicate that TCZ therapy reduced the volume of the synovium in the RA patients at three and six months after the initiation of treatment, and that MMP-3 is one of the physiological indicators of the clinical course of RA under TCZ therapy.

In the present study, the TJC values did not decrease significantly after the initiation of TCZ therapy. The average disease duration was 12.7 years, and the stage of almost all patients was classified as either Steinbrocker stage III or IV; therefore, the joint destruction (morphological changes), were significant in these patients. This is the reason why the TJC did not change even though the SUV of the joints decreased.

Concerning the other modalities used in the assessment of TCZ treatment, the usefulness of examinations with both power Doppler signals on ultrasound (US) [29] and magnetic resonance imaging (MRI) [30] have been reported. We agree that the power Doppler analyses on US help clinicians to judge the effects of TCZ treatment; however, the procedures require special skills, and in some cases, are based on subjective methods. In addition, high-resolution, contrast-enhanced MRI is limited to imaging restricted regions of the patient’s body, and to date, the scoring of MRI images is mainly subjective. In comparison, whole-body imaging analyses with FDG-PET/CT using the SUV can provide an objective method, or at the very least, an additional tool offering assistance for evaluating the efficacy of TCZ therapy.

Our joint-based analyses indicated that the SUV of bilateral knee joints had decreased at three and six months, although the swollen joint counts were not significantly decreased. In the knee joints, even if the synovitis was decreased by TCZ therapy, the joint fluid still remained a few months after the initiation of TCZ in some cases. These results may be specific for the knee joints due to their unique features.

Furthermore, in spite of the tendency for the SUV to have decreased after three and six months of TCZ therapy, statistically significant differences were observed in limited joints. On the other hand, the total SUV value (12 or eight joints) more accurately reflected the disease activity of RA patients. Therefore, adding up the SUVmax of the affected large joints is thought to be useful for assessing the efficacy of treatment in patients with RA.

There are some limitations associated with this study. First, there is limitation related to the use of the SUVmax for the follow-up, because the normal variability in FDG uptake in RA patients has not been well investigated. Additionally, the changes of the SUVmax, which occurred in the treated patients, were only partially understood. Therefore, the methodology employing the SUVmax of the affected joints was a limitation of this study. Second, we did not have an untreated comparison group in this study. Therefore, we could not completely exclude that the changes might have occurred over time without treatment. Third, although the statistical power was sufficient for the correlation analyses, which were the primary endpoint of this study, the total number of patients was small. Finally, we did not evaluate the small joints in the RA patients because such assessments require additional devices and long examination times. Further prospective studies will need to be performed to evaluate the FDG uptake in the joints of RA patients using FDG-PET/CT.

## Conclusions

In conclusion, a reduction in the FDG uptake was observed at three and six months after the initiation of TCZ therapy in the present study. The disease activity estimated on FDG-PET/CT matched the conventional parameters following the TCZ therapy in RA patients. Our results indicate that FDG-PET is one of the useful tools for monitoring the response to TCZ therapy in RA patients.

## Authors’ original submitted files for images

Below are the links to the authors’ original submitted files for images. Authors’ original file for figure 1 Authors’ original file for figure 2 Authors’ original file for figure 3
