# Supplementary figures and images for: Evaluation of tocilizumab therapy in patients with rheumatoid arthritis based on FDG-PET/CT
Source: BMC Musculoskelet Disord. 2014 Nov 22;15:393. doi: 10.1186/1471-2474-15-393 (PMC4247755; doi:10.1186/1471-2474-15-393)

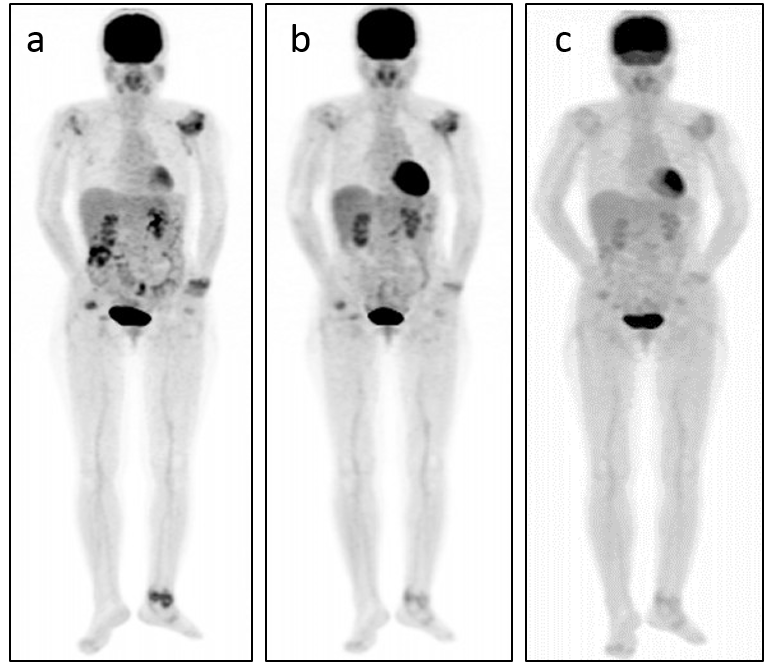

Supplement: Supplementary file 1 — Authors’ original file for figure 1 [file 12891_2014_2328_MOESM1_ESM.tiff]

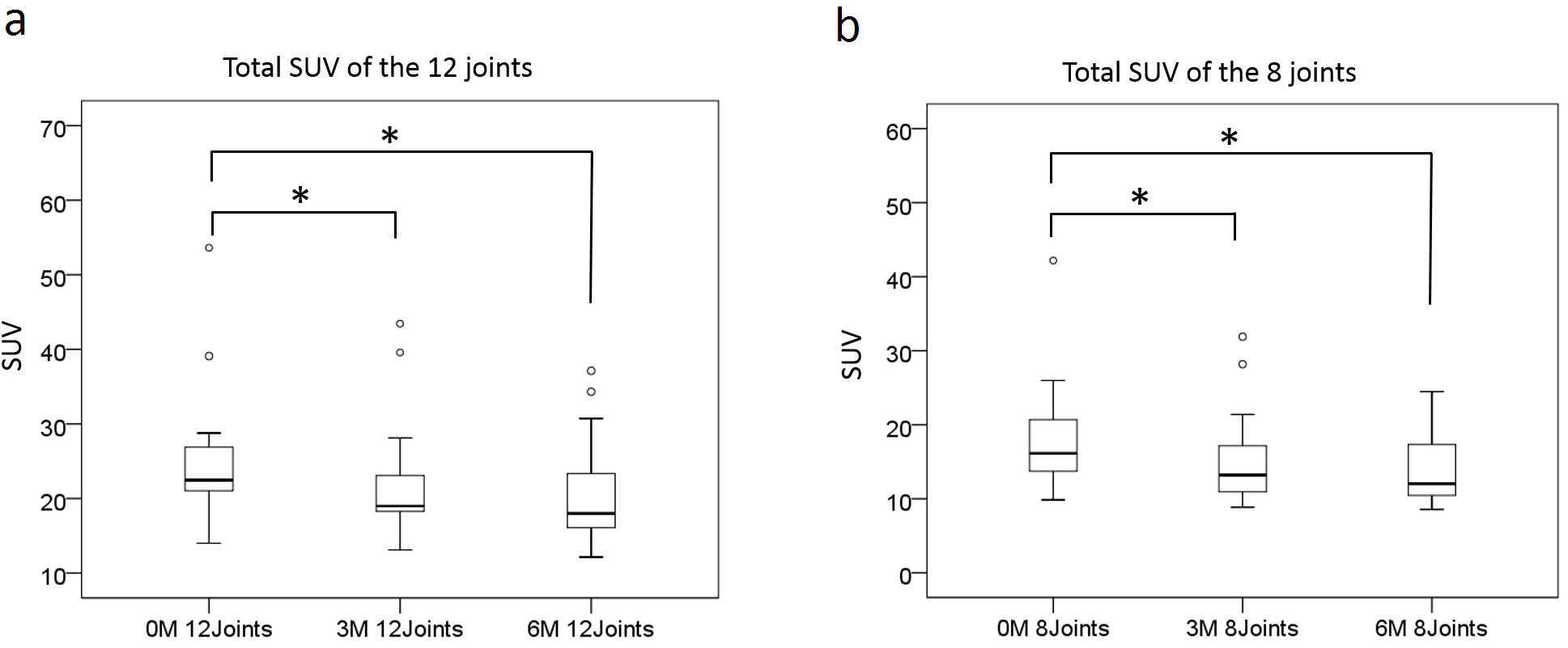

Supplement: Supplementary file 2 — Authors’ original file for figure 2 [file 12891_2014_2328_MOESM2_ESM.tiff]

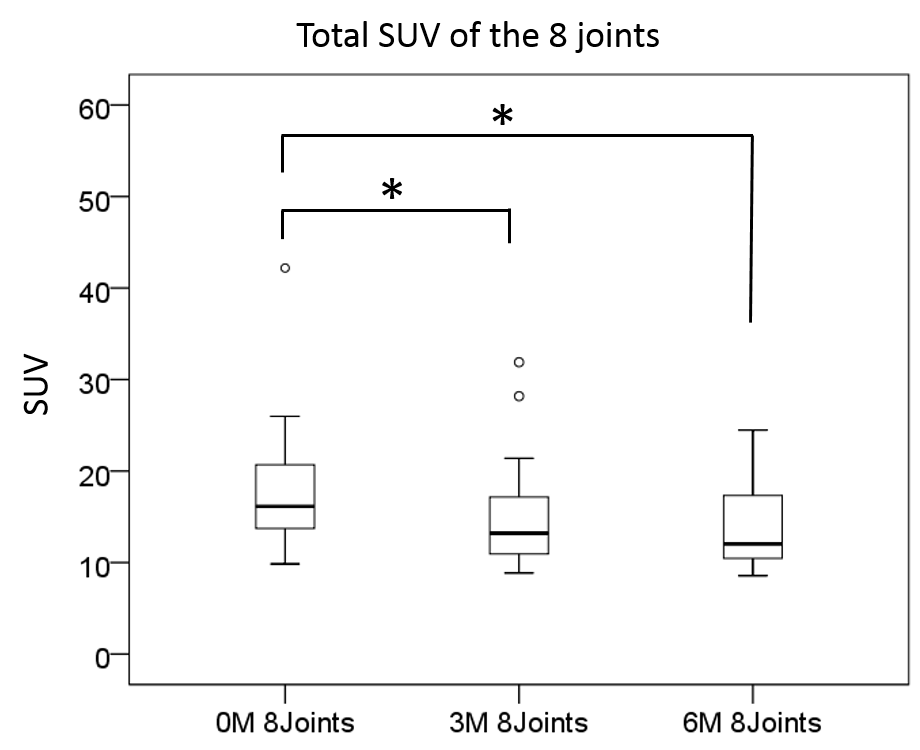

Supplement: Supplementary file 3 — Authors’ original file for figure 3 [file 12891_2014_2328_MOESM3_ESM.tiff]
